# Acute coronary syndrome due to papillary fibroelastoma detected by intravascular ultrasound imaging: a case report

**DOI:** 10.1093/ehjcr/ytaf538

**Published:** 2025-10-22

**Authors:** Hiroto Aikawa, Takayuki Yabe, Toru Kameda, Toki Toi, Takanori Ikeda

**Affiliations:** Division of Cardiovascular Medicine, Department of Internal Medicine, Toho University Faculty of Medicine, 6-11-1 Omorinishi, Ota-ku, Tokyo 143-8541, Japan; Department of Cardiovascular Medicine, Toho University Graduate School of Medicine, 6-11-1 Omorinishi, Ota-ku, Tokyo 143-8541, Japan; Division of Cardiovascular Surgery, Department of Surgery, School of Medicine, Toho University Faculty of Medicine, 6-11-1 Omorinishi, Ota-ku, Tokyo 143-8541, Japan; Department of Surgical Pathology, Omori Medical Center, Toho University, 6-11-1 Omorinishi, Ota-ku, Tokyo 143-8541, Japan; Department of Cardiovascular Medicine, Toho University Graduate School of Medicine, 6-11-1 Omorinishi, Ota-ku, Tokyo 143-8541, Japan

**Keywords:** Case report, Papillary fibroelastoma, Acute coronary syndrome, Intravascular ultrasound, Cardiac tumour

## Abstract

**Background:**

Papillary fibroelastomas (PFEs) are rare benign cardiac tumours that most commonly affect the left-sided heart valves, especially the aortic valve. Although frequently asymptomatic, PFEs can cause embolic events, arrhythmias, or coronary artery occlusion.

**Case summary:**

A 57-year-old woman with no significant medical history presented with acute chest pain and was urgently transferred to our hospital. Electrocardiography on admission showed ST-segment elevation in aVR with diffuse ST-segment depression, consistent with acute coronary syndrome (ACS). While in the emergency department, she developed a refractory ventricular fibrillation storm requiring cardiopulmonary resuscitation, intravenous amiodarone, and repeated defibrillation. Sinus rhythm was not restored, so veno-arterial extracorporeal membrane oxygenation (VA-ECMO) was initiated in the catheterization laboratory, followed by immediate coronary angiography (CAG). CAG revealed an intracoronary mass in the left main trunk (LMT). Intravascular ultrasound (IVUS) identified a heterogeneous, mulberry-like mass occluding the LMT and protruding into the aorta. Given suspicion of a soft tissue tumour near the coronary artery origin, surgical resection was performed. Histopathology confirmed the diagnosis of PFE.

**Discussion:**

This case highlights the crucial role of IVUS in diagnosing rare causes of ACS, such as PFE-related coronary occlusion. IVUS can differentiate tumour from thrombus, enabling timely surgical intervention and improving clinical outcomes.

Leaning points

- Papillary fibroelastoma (PFE) is a rare, benign cardiac tumour most often arising on left-sided valves, particularly the aortic valve.
- PFEs may cause embolic events or coronary occlusion, leading to acute coronary syndrome (ACS).
- Intravascular ultrasound is useful for identifying rare causes of ACS and differentiating tumours from thrombus.

## Introduction

Papillary fibroelastoma (PFE) is a rare, benign tumour, accounting for approximately 10% of primary cardiac neoplasms and representing the most common valvular tumour, typically arising from the aortic or mitral valve.^1^ Autopsy studies report a prevalence of <0.01% to 0.33%, with more than 80% located on left-sided valves.^1,2^ PFEs are most often diagnosed in patients aged 60–80 years, with a slight male predominance.^1^ Although frequently asymptomatic, they can cause serious complications, including systemic embolism, stroke, arrhythmias, and acute coronary syndrome (ACS) due to coronary artery occlusion.^1^ Tumour mobility has been identified as the only independent predictor of death or nonfatal embolization, underscoring the need for timely diagnosis and management.^1^ ACS caused by PFE is very rare, with only a few cases reported.^3,4^ Furthermore, diagnosis by coronary angiography (CAG) is challenging because PFEs can mimic thromboembolic disease. In this context, intravascular ultrasound (IVUS) offers high-resolution imaging and can aid in differentiating tumours from thrombi during emergency coronary interventions.

## Summary figure

**Figure:**
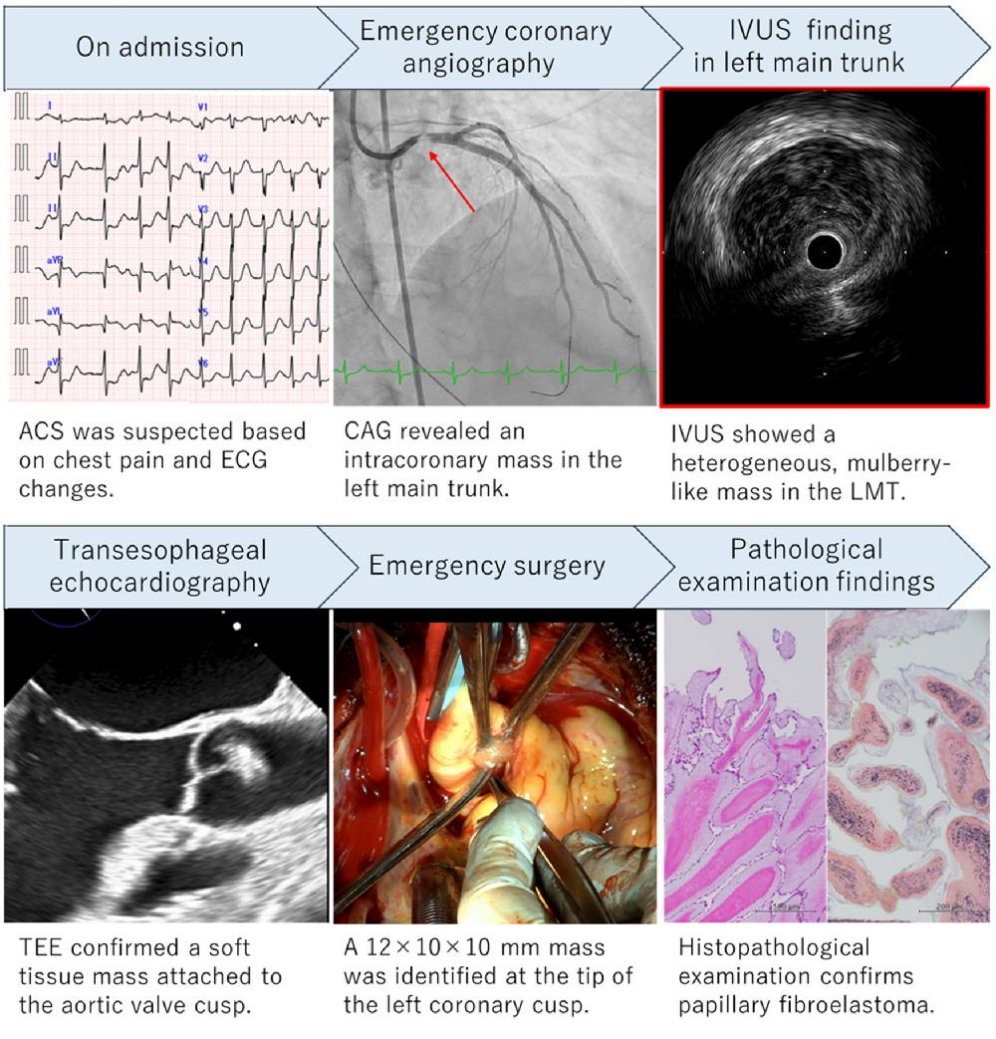


## Case presentation

**Figure 1:**
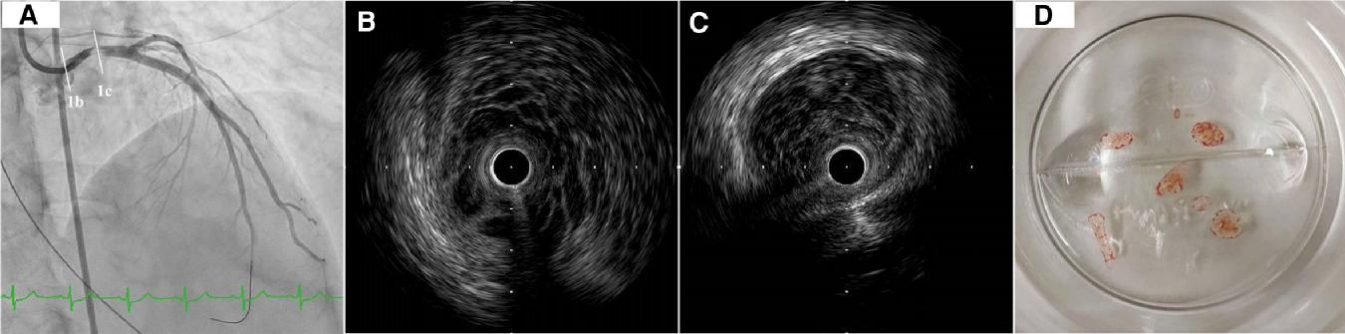
Coronary angiography, intravascular ultrasound (IVUS), and aspirated intracoronary mass findings. (*A*) Emergency coronary angiography demonstrating a mass occupying the left main coronary artery with delayed contrast filling. (*B*) IVUS demonstrating persistent protrusion of the mass from the left main trunk into the aortic root (line 1B). (*C*) IVUS demonstrating a heterogeneous, mulberry-like mass completely occluding the distal left main trunk (line 1C). (*D*) Materials aspirated using a suction catheter during thrombectomy. The fragments appeared colourless, transparent, and gelatinous.

A 57-year-old woman with no significant medical history presented with acute chest pain and was urgently transferred to our hospital. On arrival, she was in cardiogenic shock (Killip class IV) with a blood pressure of 85/48 mmHg, heart rate of 120 bpm, oxygen saturation of 89% on room air, and a lactate level of 8.7 mmol/L. Physical examination revealed cold extremities and signs of hypoperfusion. Electrocardiography demonstrated ST-segment elevation in aVR with diffuse ST depression, consistent with ACS. A bedside transthoracic echocardiogram (TTE) performed before mechanical circulatory support revealed global left ventricular hypokinesis with an ejection fraction (EF) of approximately 30%, without intracardiac masses. During initial evaluation, the patient developed recurrent ventricular fibrillation (VF). Immediate cardiopulmonary resuscitation, intravenous amiodarone, and repeated defibrillation were required. Due to haemodynamic instability, detailed echocardiographic evaluation was not feasible. Veno-arterial extracorporeal membrane oxygenation (VA-ECMO), endotracheal intubation, and mechanical ventilation were instituted for stabilization before urgent transfer to the catheterization laboratory. CAG revealed an obstructive intracoronary mass within the left main trunk (LMT), causing markedly delayed contrast filling (*Figure 1A*). After successful wiring of the left anterior descending artery, IVUS demonstrated a heterogeneous, mulberry-like mass completely occluding the LMT (*Figure 1B, C*). Aspiration thrombectomy (*Figure 1D*) and balloon angioplasty with a perfusion balloon were performed, partially restoring coronary perfusion. Subsequent IVUS showed persistent protrusion of the mass from the LMT into the aortic root (*Figure 1B*), raising suspicion of a soft tissue tumour originating from the coronary ostium.

**Figure 2:**
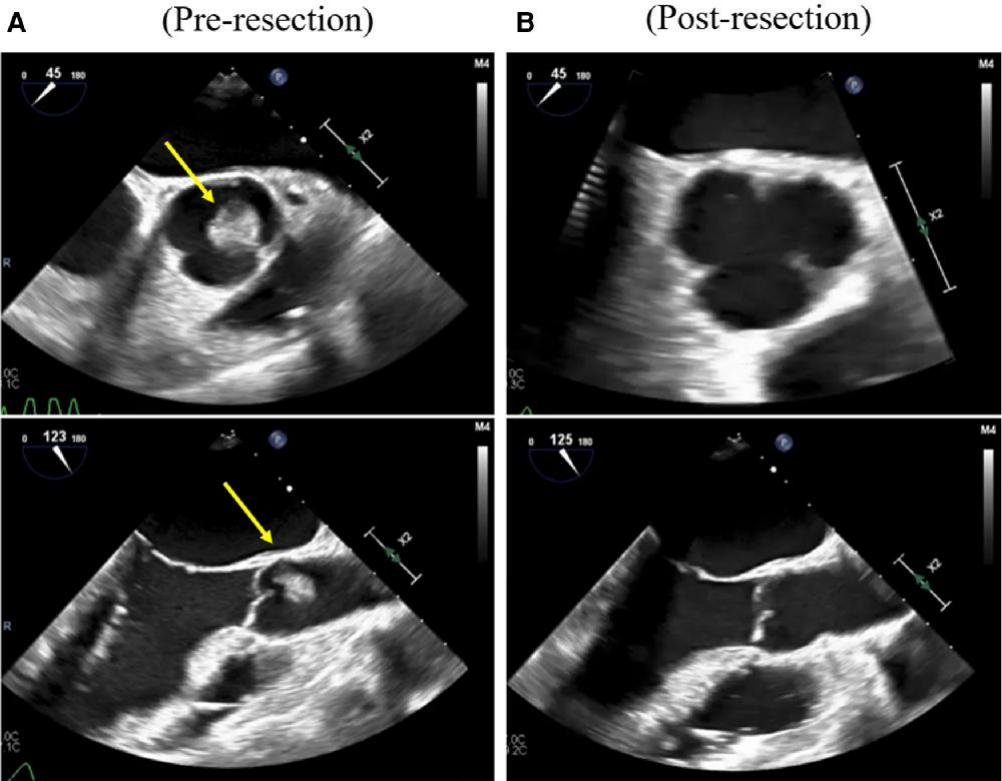
Intraoperative transoesophageal echocardiography (TEE) findings. (*A*) Intraoperative TEE identifying a soft tissue mass attached to the left coronary cusp of the aortic valve (arrow). (*B*) Postoperative TEE confirming successful tumour resection with preserved aortic valve function.

**Figure 3:**
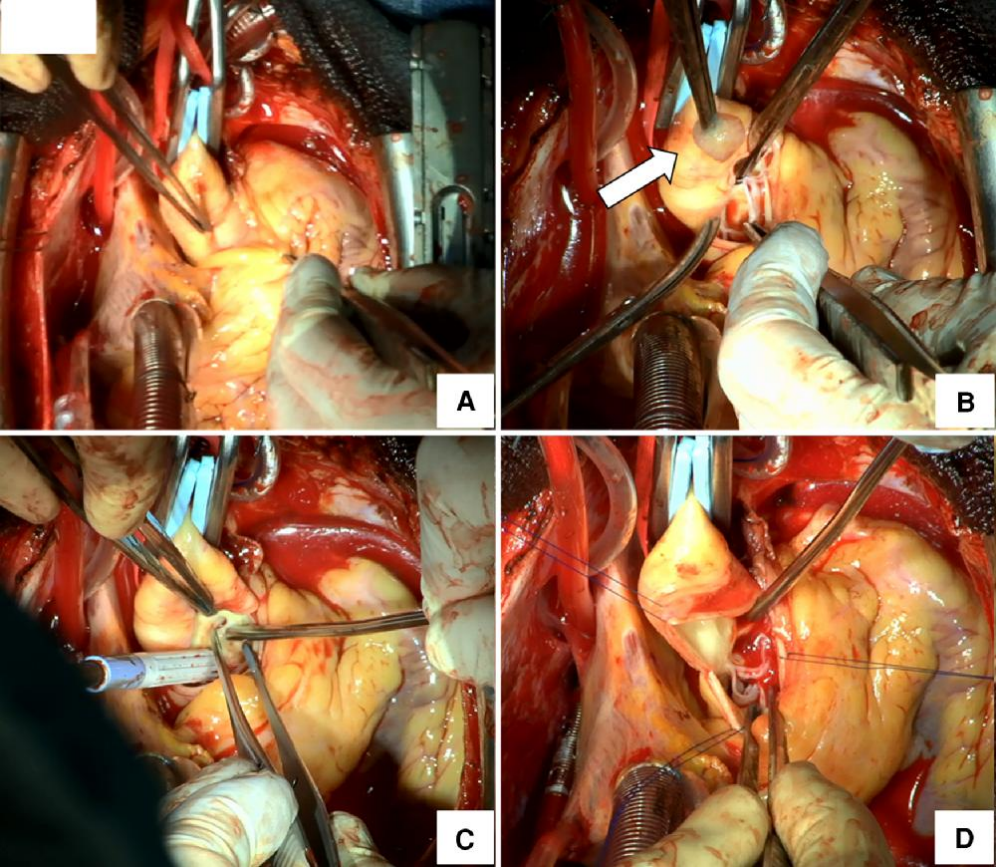
Intraoperative surgical views and gross pathology. (*A*) Median sternotomy with transverse aortotomy exposing the aortic root. (*B*) Gross specimen of the excised mass (12 × 10 × 10 mm) arising from the tip of the left coronary cusp (arrow). (*C*) Post-resection inspection of the aortic valve confirming absence of residual tumour and intact leaflet mobility. (*D*) Closure of the aortotomy and completion of chest closure.

**Figure 4:**
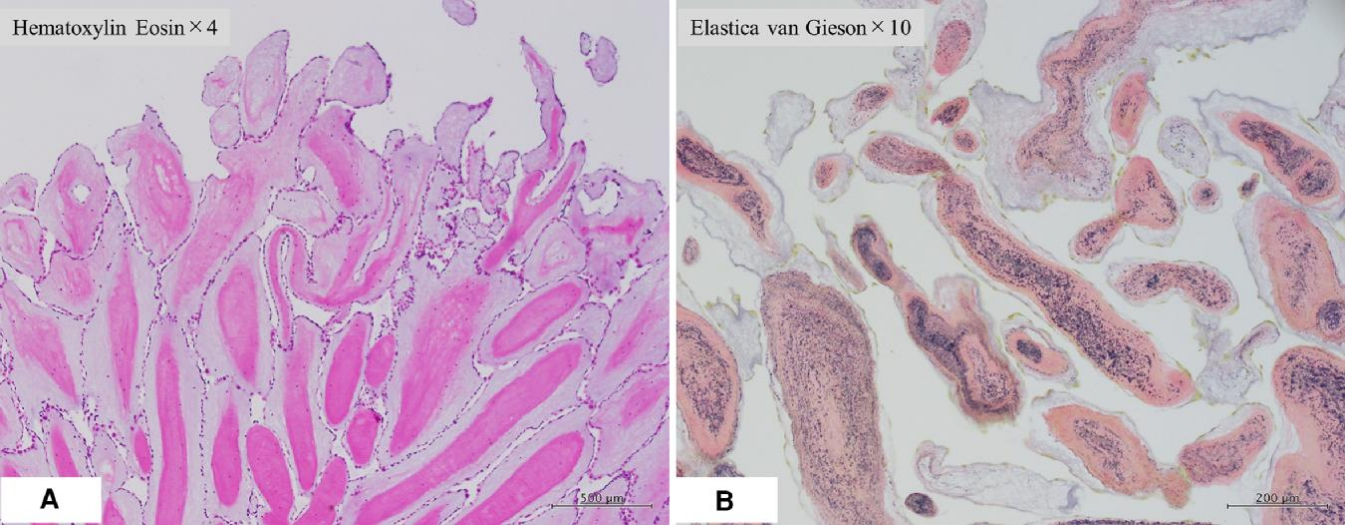
Histopathological examination. (*A*) Haematoxylin and eosin (H&E) staining, × 4 magnification, demonstrating multiple papillary projections. (*B*) Elastica-Van Gieson staining, × 10 magnification, confirming collagen and elastic fibre composition. These features are consistent with papillary fibroelastoma.

The patient was referred for emergency surgery. Intraoperative transoesophageal echocardiography (TEE) identified a mobile soft-tissue mass attached to the left coronary cusp of the aortic valve (*Figure 2*). Surgical inspection revealed a 12 × 10 × 10 mm pedunculated mass arising from the tip of the left coronary cusp (*Figure 3*). Histopathological examination demonstrated multiple papillary fronds composed of collagen and elastic fibres, consistent with PFE (*Figure 4*). The patient was weaned from VA-ECMO and mechanical ventilation by postoperative day 7. She received aspirin (100 mg daily) and bisoprolol (2.5 mg daily) during hospitalization. TEE before discharge showed near-complete recovery of left ventricular function (EF: 50%) and confirmed complete tumour resection. As cardiac function had normalized, both medications were discontinued. She was discharged on postoperative day 42 without complications, with plans for outpatient follow-up and annual echocardiography. Her recovery was uneventful.

## Discussion

The main limitation of this report is its description of a single case, which restricts generalizability. PFEs are rare, benign cardiac tumours that predominantly affect left-sided heart valves, particularly the aortic valve.^1,2^ Histologically, PFEs are characterized by narrow, elongated papillary fronds composed of mucopolysaccharides, elastic fibres, and spindle-shaped cells resembling fibroblasts or smooth muscle cells.^5^ In this case, histopathology revealed elongated polypoid projections with collagen and elastic fibres, consistent with PFE.

The differential diagnosis of valvular masses is broad. Infective endocarditis should be considered in patients with fever or signs of infection. Other possibilities include thrombus, Lambl’s excrescences, and valvular calcifications. Rarely, myxomas, malignant tumours, or metastases may also involve the aortic valve.^6,7^ Although often asymptomatic, PFEs can cause severe complications such as embolic events, arrhythmias, or coronary occlusion.^1^ Prognosis after resection is favourable because of the minimal risk of recurrence. In asymptomatic patients, tumour size >9 mm, high mobility, or independent motion are predictors of adverse events and may justify prophylactic surgery.^1^

In 2010, Raju *et al*. reported a case of LMT embolism caused by PFE treated with valve-preserving surgery.^8^ The patient initially received anticoagulation based on angiographic suspicion of thromboembolism, but later required surgery after echocardiography revealed a valve mass. More recently, two reports have described the use of IVUS in PFE-associated ACS.^9,10^ In Tsumaru *et al*., the patient experienced two life-threatening arrhythmic episodes, with diagnosis and surgery occurring on the second admission.^9^ The initial presentation required percutaneous cardiopulmonary support, but the causal link to PFE was unclear. This case highlights that, while echocardiography remains the gold standard, it may be insufficient in acute settings.

In Kobayashi *et al*., IVUS findings closely resembled ours, demonstrating a heterogeneous soft-tissue mass extending towards the sinus of Valsalva from the LMT.^10^ These findings emphasize the value of IVUS in delineating lesion morphology and extent during the procedure. Differentiating tumour from thrombus is critical (*Table 1*). Thrombi typically appear as low-echogenic, irregular masses, often with a layered or lobulated appearance and microchannels or speckled echogenicity on IVUS,^11^ whereas soft-tissue tumours such as PFE exhibit heterogeneous internal architecture without marked posterior acoustic attenuation. In our case, the absence of high attenuation and the mulberry-like heterogeneous pattern supported the diagnosis of PFE, prompting urgent surgical resection.

**Table 1:** Intravascular ultrasound characteristics of papillary fibroelastoma and thrombus

Feature	Papillary fibroelastoma	Thrombus
Echogenicity	Heterogeneous	Predominantly low
Surface texture	Frond-like, mobile	Irregular or amorphous, mobile
Posterior acoustic attenuation	Absent	Present
Attachment site	Primarily to the valve cusp	Typically, free-floating
Response to aspiration	Minimal reduction in size	Often decreases in size
Response to plain old balloon angioplasty	No compression	Compression observed

ACS in our patient resulted from LMT occlusion by a PFE arising from the aortic valve, and IVUS was essential for characterizing the mass and guiding surgery. Bedside TTE also remains essential in shock, enabling rapid assessment of ventricular function and sometimes mass detection. Ideally, such imaging may facilitate direct transfer to surgery, but in this case, refractory VF precluded detailed evaluation.

This case underscores the value of IVUS in diagnosing rare causes of ACS, particularly when angiographic findings are atypical. By offering high-resolution cross-sectional imaging, IVUS helps distinguish thrombus from tumour and expedites definitive management. To our knowledge, this is among the few reports of IVUS-confirmed PFE causing ACS due to LMT occlusion, highlighting its diagnostic and clinical utility. In conclusion, this case represents a rare presentation of ACS caused by PFE and emphasizes the importance of multimodal imaging, particularly IVUS, for timely diagnosis and management.

## Data Availability

The data supporting this article will be made available upon reasonable request to the corresponding author.

## Lead author biography

Hiroto Aikawa is a 12th-year physician at Toho University Medical Centre Omori Hospital, specializing in Internal Medicine and Cardiology.

## Notes

**Consent:** The authors confirm that written consent for submission and publication of the clinical case, including images and associated text, has been obtained from the patient in line with the COPE guidance.

**Funding:** No funding was provided for this work.
